# Resource utilization and costs associated with the addition of an antimuscarinic in patients treated with an alpha-blocker for the treatment of urinary symptoms linked to benign prostatic hyperplasia

**DOI:** 10.1186/s12894-017-0275-6

**Published:** 2017-09-12

**Authors:** Antoni Sicras-Mainar, Ruth Navarro-Artieda, Ana Mª. Mora, Marta Hernández

**Affiliations:** 1 Dirección de Planificación, Badalona Serveis Assistencials SA, Via Augusta, 9, 08911 Badalona, Barcelona, Spain; 2 Documentación Médica, Hospital Germans Trias i Pujol, Badalona, Barcelona, Spain; 3 Medical Department, Astellas Pharma S.A, Madrid, Spain

**Keywords:** Lower urinary tract symptoms, Benign prostatic hyperplasia, Antimuscarinics, Resource utilization, Costs

## Abstract

**Background:**

There has been a change in the focus of attention from prostate to bladder, as the etiology of lower urinary tract symptoms (LUTS) makes the bladder an additional therapeutic target. This study aims to evaluate the use of resources and costs associated with the addition of an antimuscarinic (AM) in patients receiving an alpha-adrenergic-blocker (AAB) for the treatment of LUTS linked to benign prostatic hyperplasia (BPH).

**Methods:**

A multicentre, retrospective study was conducted using patient records from the databases of six primary care centers in Spain. Men with moderate-to-severe LUTS (IPSS > 7) who were initiated on AM treatment between January 2010 and December 2012 without previous treatment with an AM or 5-alpha reductase inhibitor (5-ARI) and had been on treatment with an AAB for a minimum of 6 months prior to the addition of the AM with a minimum of two records in the database were included. Comorbidity, treatment persistence, and use of resources and costs (direct and indirect) during monotherapy (AAB alone) and following the introduction of combination therapy (AAB + AM) over a treatment period of up to a year were compared. A paired sample Student t-test was performed where *p* < 0.05 were considered significant.

**Results:**

One hundred and ninety-one patients (mean age (SD): 70 (10.4) years) were treated with combination therapy. Treatment persistence on combination therapy after 12 months was 65.4% (95% CI: 58.8-72.2%). Use of resources was numerically lower after initiation of combination therapy vs pre-treatment (AAB alone) period for medical visits (/year/patient) (13.4 (4.6) vs 15.4 (4.4) *p* < 0.010), percentage of patients using concomitant medication (13.3% vs 19.1%) and use of pads (9.7% vs 13.4%) among others analyzed. Comparing AAB vs AAB + AM, there were a numeric reduction in total cost/year (€2399 vs €2011; *p* = 0.135) and a reduction of costs due to medical visits (€645 vs. €546; *p* = 0.003) and concomitant medication (€181 vs. €101; *p* = 0.009).

**Conclusions:**

The addition of an AM agent in patients treated for LUTS with AAB is associated with a lower use of healthcare resources in terms of number of medical visits, and concomitant medications required, thereby leading to reduction of overall costs to the healthcare system.

## Background

Benign prostatic hyperplasia (BPH) is a common condition and a major source of morbidity in older men [1–3]. Its prevalence increases with age, and it presents in more than 50% of men above 50 years old and in up to 80% of males above 90 years old [4–6]. The *International Continence Society* proposed the term Lower Urinary Tract Symptoms (LUTS) to describe symptoms associated with the storage and voiding phases of the urinary cycle [1]. Male LUTS have traditionally been attributed to the prostate, but breakthroughs in the knowledge of *bladder dysfunction* and its physiopathology have led to the understanding that the storage symptoms of male LUTS may be due to co-existing overactive bladder or bladder malfunction secondary to bladder outlet obstruction of prostatic origin [1, 3, 7].

The insufficient response in alleviating LUTS related to BPH in some cases, together with the increasing recognition of the complexity of the pathophysiology of the lower urinary tract as a functional unit, has helped to shift the focus of attention from the prostate to the bladder as a possible cause of LUTS, thus making it an additional therapeutic target [8–10]. This change of perspective, acknowledging the multifactorial etiology of male LUTS, and accepting that not all of the symptoms are necessarily related to the prostate, is mirrored in the current guidelines of Scientific Societies [1, 3, 5–7]. The main treatment objectives are to reduce symptoms, improve quality of life and prevent the development of complications [1, 2, 7–10]. In this regard, and as a step prior to treatment selection, the severity of symptoms should be assessed with the International Prostate Symptom Score (IPSS) [11, 12]. Drug treatment is indicated in patients with moderate-to-severe symptoms who do not present an absolute indication for surgery [13], and the combination of an antimuscarinic with an alpha-blocker is justified in patients with BPH and LUTS compatible with co-existing overactive bladder [7, 9, 10, 13, 14]. There are several reasons that have led to this treatment rationale, particularly: a) the profile of patients with mixed storage and voiding LUTS with an important storage symptom component; b) the recommendations of the EAU guidelines [1]; and c) the scientific evidence on the effectiveness of alpha-blocker and antimuscarinic combination in this patient population. However, there is limited data on the impact these treatments have on resource utilization and costs.

The limited evidence in clinical practice available on the combined use of alpha-blockers and antimuscarinics in patients with LUTS and its impact on resource utilization and costs makes this study particularly relevant. The main objective of the study was to compare the level of resource utilization and both direct and indirect costs before and after the addition of an antimuscarinic in patients receiving an alpha-blocker for the treatment of LUTS associated with BPH in routine clinical practice.

## Methods

### Study design and population

This was an observational, multicentre, retrospective study on the use of resources and costs associated with lower urinary tract symptoms(LUTS) suggestive of benign prostatic hyperplasia (BPH) according to different clinical profiles in routine clinical practice. This study was based on a review of computer-based medical records of patients identified from the databases of six primary care centers [PCC] managed by Badalona Serveis Assistencials SA. Information on secondary care health resource utilization by these patients was obtained from the two reference hospitals: The Municipal Hospital and Hospital Universitari Germans Trías i Pujol de Badalona (specialized care).

### Inclusion and exclusion criteria

All male patients suffering from LUTS who started add-on therapy with an AM between January 2010 and December 2012 were included in the study if they fulfilled the following criteria: a) aged ≥45 years; b) assigned to the geographical reference area; c) no previous treatment with AM or 5-ARI; d) current treatment with an AAB (for a minimum of six months prior to the addition of the AM); e) moderate-to-severe LUTS (IPSS > 7); f) regular follow-up was likely (defined as having presented two or more times according to health records), and g) on a chronic treatment prescription program with a proven record of the daily dose, timeframe and the duration in each administered treatment. The following patients were excluded: a) those transferred and/or moved from other geographic areas; b) permanently institutionalized patients; c) patients with grade III-IV prostate volume by digital rectal examination (DRE) (>40 ml); and d) coexistence of other urological conditions (prostate, bladder or kidney cancer, chronic urinary tract infection, calculi, urethral stricture, chronic pelvic pain syndrome and pelvic organ surgical record). The index date was the date the patient started on an antimuscarinic and the follow-up was one year from the index date.

### Treatment description and persistence

Patients being treated for BPH were identified according to the Anatomical Therapeutic Chemical Classification System (ATC) code G04C [15]. The choice of medication for a specific patient was based on the doctor’s judgment during clinical practice. Persistence was defined as the time, measured in months, without the patient dropping out of the initial treatment or without switching to another medication at least 30 days after the initial prescription. A patient was classified as being persistent if they had no treatment discontinuation or switch to another medication during the 12-month follow-up period.

### Identification of patients with LUTS associated with BPH

The diagnosis of BPH was based on the International Classification of Primary Care (ICPC-2), component 7, diseases and health problems [16] (Y85), and the hospital discharge and emergency coding according to the International Classification of Diseases, 9th Revision, Clinical Modification; ICD-9-MC [600]). The following disease-related variables were also obtained from the database records: a) years of disease progression (BPH); b) symptom profile (storage, voiding and post-micturition symptoms); c) score on the IPSS scale, d) prostate volume: volume I (<20 ml) and volume II (20-40 ml) by DRE; e) body mass index (BMI, kg/m^2^) and other lab test parameters (systolic and diastolic blood pressure (mmHg), total cholesterol (mg/dL) and serum creatinine (mg/dL)) f) prostate-specific antigen (PSA) (ng/mL); and g) concomitant medication related with LUTS (antidepressants, anxiolytics and antibiotics).

### Sociodemographic and comorbidity data

Patient demographics including age, occupational status, and concomitant diseases were assessed. The level of comorbidity was assessed based on the Charlson comorbidity index [17], which measures the level of severity of the patient’s conditions; and the number of chronic conditions. Comorbidity was adjusted using the Adjusted Clinical Groups (ACG) system, which is a patient classification system that measures the expected-consumption of healthcare resources according to a particular disease pattern, age and sex [18]. The ACG application provides the resource utilization bands (RUB), and each patient, depending on their general morbidity, is grouped into one of the 5 mutually exclusive categories (1: healthy or very low morbidity users, 2: low morbidity, 3: moderate morbidity, 4: high morbidity, and 5: very high morbidity).

### Use of resources and cost analysis

Direct healthcare costs were those associated with BPH-related healthcare activity by health professionals (primary care visits, secondary care visits and hospital emergencies, days of hospitalization, emergencies and diagnostic and/or therapeutic requests). Indirect costs related to lost occupational productivity (days off work) were also obtained. The cost was expressed as mean cost per patient and year. The different study concepts and their economic evaluation are detailed in Table 1 (corresponding to 2012). The different rates were obtained from the centers’ analytical accounting, except medication and days off work. Medical prescriptions were quantified according to the recommended retail price on the container at the time of prescription. The cost of days off work or lost activity was quantified according to the mean interprofessional wage (source: National Statistics Institute-INE) [19]. This study did not provide for the calculation of non-health direct costs, i.e. those regarded as “out-of-pocket expenses” or paid for by the actual patient/family, as they are not recorded in the database and direct access to the patient was not possible. Cost calculation was based on the use of pads for urinary incontinence and the concomitant medication associated with the clinical consequences of BPH. Costs were determined during the 12 months prior to (pre-treatment) and the 12 months following (treatment) the date that the AM add-on therapy was started (index date).

**Table 1:** Breakdown of unit costs and lost occupational productivity (2012)

Health and non-health resources	Unit costs (€)
Medical visits	
Primary care medical visit	23.19
Hospital emergency medical visit	117.53
Hospitalization (day)	380.00
ICU/coronary hospitalization (day)	850.00
Specialized Care medical visit	104.41
Complementary tests	
Laboratory Tests	22.30
Radiology^a^	18.50
Diagnostic/therapeutic tests^b^	97.12
Pharmaceutical prescription^c^	RRP VAT
Occupational productivity – indirect costs	
Cost per day not worked^d^	101.21

Source of the health resources: own analytical accounting

Values expressed in Euros (€). RRP VAT: Recommended retail price plus VAT; ICU: Intensive Care Unit

^a^Includes: simple conventional radiology

^b^Includes: radiology with contrast, ultrasound, TC scan, NMR, uroflowmetry, cystoscopy, cytology

^c^Includes: pads for incontinence and concomitant medication (anxiolytics, antidepressants and antibiotics)

^d^Source: National Statistics Institute (INE)

### Confidentiality of information

Confidentiality of medical records of patients identified from the databases was observed in agreement with Spanish Data Protection Law. The study was classified by the Spanish Agency for Medicines and Medical Devices (EPA-OD) and was subsequently approved by the Clinical Research Ethical Committee of the Hospital Universitari Germans Trías y Pujol de Badalona.

### Statistical analysis

The mean, standard deviation and 95% confidence intervals (CI) were produced for normally distributed variables; and median and interquartile intervals (percentiles) for other variables. The Student t-test (for paired groups) was used to compare pretreatment versus combination treatment period, and Pearson’s linear correlation according to data distributed and calculated for all the variables.

The comparison of the use of resources and their corresponding costs, between the first period of treatment with AAB and the second period after starting add-on therapy (AAB + AM), was performed according to Thompson and Barber’s (2000) recommendations [20]^,^ using a general linear model (analysis of covariance -ANCOVA-). Continuous and categorical independent variables: age, RUB, the Charlson index and number of years of disease evolution were included in the model as covariables (procedure: estimated marginal means; Bonferroni correction).

Data was presented as adjusted mean differences between treatments with the corresponding 95% confidence intervals calculated, using re-sampling techniques (bootstrapping) corrected for bias, given the non-normal distributions of the variables with respect to resource utilization and costs. All data were entered and analyzed in the SPSSWIN statistics application, version 17 Safety.

This was a non-interventional study. According to the regular clinical practice of the participating physicians, suspected adverse reactions detected during the course of the study had to be reported by the investigators as promptly as possible to the competent authority in matters of pharmacovigilance of the Autonomous Community corresponding to the healthcare area. For this purpose, the individual reporting form for suspected adverse reactions (“yellow card”) had to be used, following article 7 of Law 1344/2007, of 11 October.

During the conduct of the study, the sponsor was not made aware of any potential safety information.tics applicationtics application

## Results

**Fig. 1:**
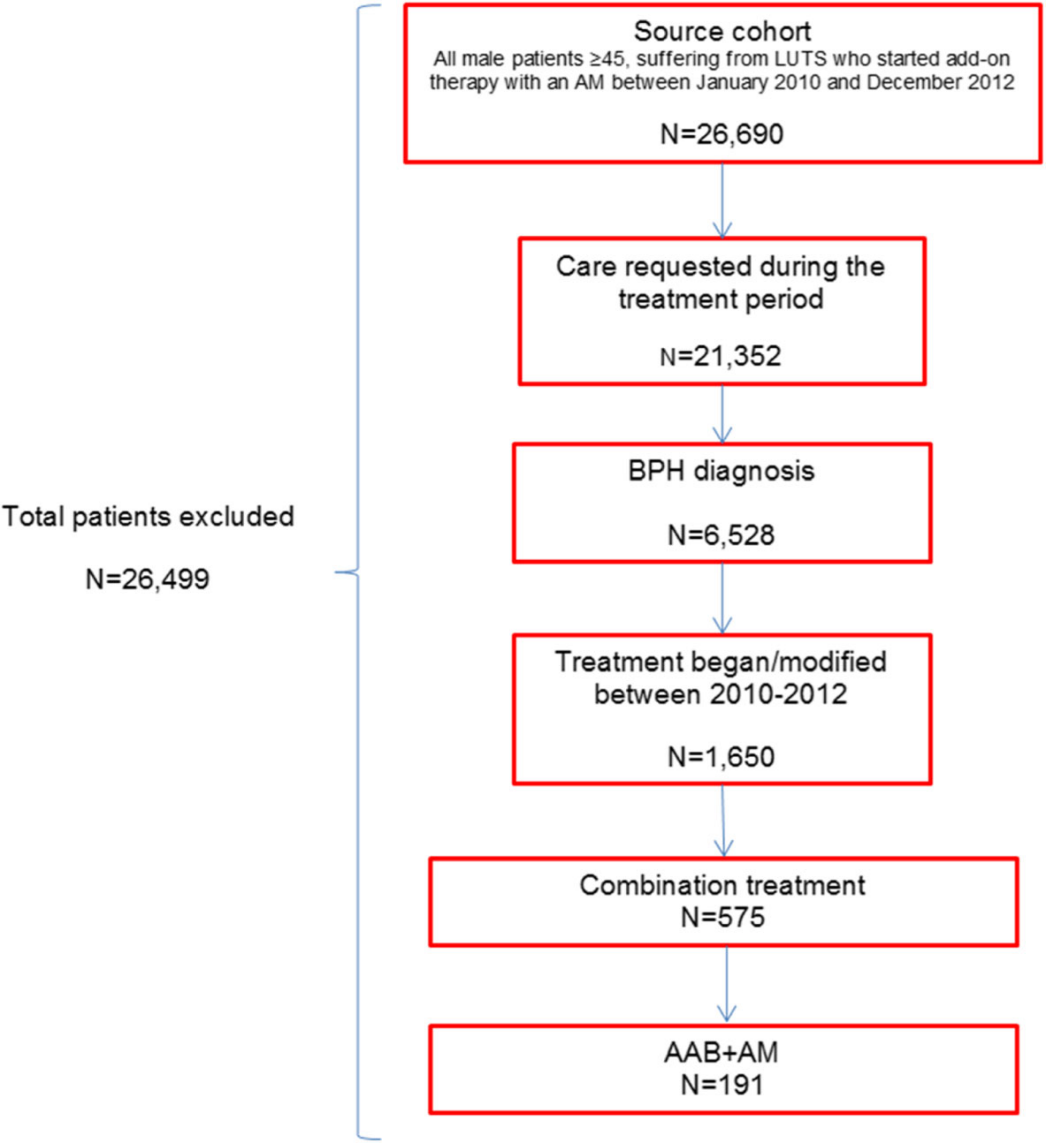
Patients included in the study. AAB: Alpha -blocker; AM: Antimuscarinic, LUTS: Lower Urinary Tract Symptom

The population from the database was comprised of 26,690 subjects ≥45 years of age, 21,352 of whom requested care during the recruitment period. A total of 6528 patients were diagnosed with BPH. Between 2010 and 2012, 1650 patients began/modified treatment, 575 of whom initiated combination treatment (AAB + AM). Of these, 66.8% (*n* = 384) were given treatment with an AAB and 5-ARI, and 33.2% (*n* = 191) with an AAB and AM (Fig. 1).

Table 2 shows the baseline characteristics, the clinical profile (LUTS) and the biochemical and anthropometric parameters of the 191 patients (mean age 70.0 (10.4)) receiving an AAB and AM combination. Patients receiving a combination of AAB and AM, had a high predominance of storage symptoms (87.4%) and post-micturition symptoms (91.1%), and co-existence of voiding symptoms in (47.6%). There was also a high percentage of patients with prostate volume lower than 20 cm^3^ (93.2%), a low percentage of patients with a prostate volume 20-40 cm^3^ (6.8%) and a high percentage of patients with PSA <1.5 ng/ml (79.3%) in those receiving a combination of AAB and AM.

**Table 2:** Baseline characteristics of the population on combination (AAB + AM)

Subjects	N = 191
Demographic characteristics	
Mean age, years	70.0 (10.4)
Ranges: 45-64 years	29.8%
65-74 years	33.5%
≥ 75 years	36.6%
Pensioner	88.0%
General comorbidity	
Number of diagnoses	7.7 (4.2)
Charlson Comorbidity Index	0.6 (0.7)
RUB (morbidity)	3.0 (0.7)
1 (very low)	4.7%
2 (low)	10.1%
3 (moderate)	69.2%
4 (high)	15.4%
5 (very high)	0.6%
Specific comorbidity	
Arterial hypertension	59.8%
Dyslipidemia	51.3%
Cardiovascular event	32.8%
Diabetes mellitus	27.5%
Active smoker	22.2%
Vasculocerebral accident	22.2%
Organ failures	19.6%
COPD	18.5%
Obesity	18.0%
Ischemic heart disease	17.5%
Depressive syndrome	14.8%
Malignant neoplasms	13.2%
Alcoholism	5.3%
Asthma	4.8%
Neuropathies	4.8%
Dementias	2.6%
Clinical profile (symptoms)	
Storage	87.4%
Voiding	47.6%
Post-micturition	91.1%
Prostate volume (%)	
Volume I (<20 ml)	93.2%
Volume II (>20-40 ml)	6.8%
IPSS scale score	
Moderate symptoms (8-19 points)	71.2%
Severe symptoms (20-35 points)	28.8%
Biochemical/anthropometric parameters	
Systolic blood pressure, mmHg	129.4 (15.0)
Diastolic blood pressure, mmHg	72.5 (10.6)
Body mass index kg/m^2^	29.0 (4.0)
Total cholesterol, mg/dL	186.6 (42.1)
Serum creatinine, mg/dL	1.1 (0.4)
PSA (ng/mL)	0.9 (0.9)
Low PSA (<1.5 g/L)	79.3%
High PSA (≥1.5 g/L)	20.7%

Values expressed as a percentage or mean (standard deviation)

*AAB* alpha-blocker, *AM* antimuscarinic, *COPD* chronic obstructive pulmonary disease, *IPSS* international prostate symptom score, *PSA* prostate-specific antigen, *RUB* resource utilization bands

According to the IPSS, 71.2% of those receiving an AAB plus AM presented moderate symptoms (8-19 points) and 28.8% presented severe symptoms (20-35 points). The most frequent comorbidity reported in this patient population was arterial hypertension (59.8%).

Table 3 shows the treatment description and persistence rate of the 191 subjects analyzed in this study. The mean (SD) duration of the treatment with single therapy (AAB) prior to adding on AM was 15.7 (13.5) months. Treatment persistence on combination therapy (AAB + AM) after 12 months was 65.4%.

**Table 3:** Description and persistence of the treatment administered from the series studied (CI 95%)

Subjects	N = 191
Time since diagnosis, years	
Mean (SD)	8.0 (3.7)
Median (P25 - P75)	9.0 (4.5-10.5)
Duration of the treatment (single therapy), months	
Mean (SD)	15.7 (13.5)
Median (P25 - P75)	14.1 (2.0-23.3)
Duration of the treatment (double therapy), months	
Mean (SD)	10.2 (7.1)
Median (P25 - P75)	9.0 (4.0-11.5)
Treatment persistence^a^	
Persistence on double therapy, 12 months	65.4%
CI 95%	58.8-72.2%

Values expressed as mean (SD: standard deviation) or percentage, P: percentiles

^a^ *Persistence* was defined as the time, measured in months, without the patient dropping out of the initial treatment or without switching to another medication at least 30 days after the initial prescription. A patient was classified as being persistent if they had no treatment discontinuation or switch to another medication during the 12-month follow-up period. CI: Confident Interval

**Fig. 2:**
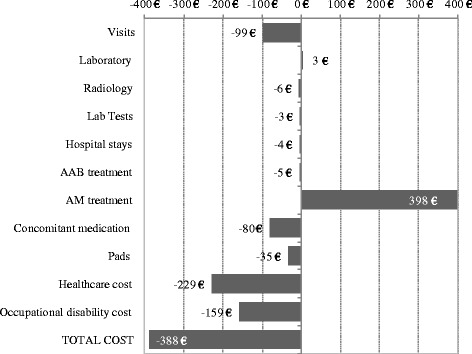
Differences in mean cost after adding an AM agent. Values in Euros (€); AAB:Alpha-blockers; AM: Antimuscarinics

The comparison of pre-treatment (AAB alone) and combination treatment (AAB + AM) healthcare resource use and costs per patient per year is shown in Table 4. Use of resources was lower after initiation of AM + AAB vs pre-treatment (AAB alone) period for medical visits in general (13.4 (4.6) vs 15.4 (4.4) *p* < 0.010), primary care (10.6 (7.3) vs 12.1 (7.5) and hospital emergency visits (0.4 (0.8) vs 0.7 (0.7)) (p < 0.010 both). Concomitant medication (13.3% vs 19.1%) and use of pads (9.7% vs 13.4%) present a numerical (not statistically significant) reduction in use of resources with AAB + AM versus AAB alone. During the combination treatment period (AAB + AM), 84.3% of the total cost was healthcare related vs 80.2% on the pre-treatment period (AAB alone) and 15.7% was non-healthcare related vs 19.8% on the pre-treatment period (AAB alone). During the pre-treatment period (AAB alone), the total cost per patient per year was €2399 versus €2011 in the treatment period, despite including the cost of the AM. However, this numerical difference was not statistically significant (*p* = 0.135). A reduction in cost was also shown from the pre-treatment (AAB alone) to the treatment period (AAB + AM) for medical visits (from €645 to €546 (*p* < 0.01)) and concomitant medication (from €181 to €101 (*p* < 0.001)). Differences in mean costs are shown in Fig. 2.

**Table 4:** Use of resources and healthcare costs per patient/year of the series studied

Periods	Pretreatment (AAB)	Treatment^a^ (AAB + AM)
Use of resources		
Medical visits (all)	15.4 (4.4)	13.4 (4.6)^†^
- Primary Care	12.1 (7.5)	10.6 (7.3)^†^
- Specialists	2.6 (4.3)	2.4 (3.0)
- Hospital emergencies	0.7 (0.7)	0.4 (0.8)^‡^
Laboratory	1.4 (1.6)	1.6 (1.7)
Radiology	1.0 (1.9)	0.7 (1.2)
Complementary tests	0.3 (0.9)	0.2 (0.5)
Concomitant medication^b^	19.1%	13.3%
Pads	13.4%	9.7%
Hospital stays	2.0 (7.7)	2.0 (6.1)
Occupational disability, days	4.7 (17.9)	3.1 (17.1)
*Costs (in €)*		
Medical visits (all)	645 (460)	546 (420)^†^
Laboratory	32 (55)	35 (39)
Radiology	19 (25)	13 (22)
Complementary tests	20 (42)	17 (46)
Hospital stays	750 (2.449)	746 (2.337)
Specific medication		
Alpha-adrenergic blockers	195 (174)	190 (159)
Antimuscarinics	–	398 (117)
Concomitant medication^b^	181 (99)	101 (75) ^‡^
Pads	82 (144)	47 (138)
Healthcare cost	1924 (2478)	1695 (2542)
Occupational disability cost	475 (1677)	316 (1744)
Total cost	2399 (3113)	2011 (3026)

Values expressed as mean (SD: standard deviation) or percentage, p: statistical significance

*AAB* alpha-blocker, *AM* antimuscarinic, *€* euros

^a^Period of combined treatment (12 months after the index date)

^b^Concomitant medication (anxiolytics, antidepressants and antibiotics)

Non-statistically significant difference when it is not indicated between the comparison by pairs (pretreatment vs. treatment), ^‡^p < 0.001, ^†^p < 0.01

## Discussion

This study using data from the Spanish National Health System shows that patients with LUTS associated with BPH who were on treatment with an AAB and initiate a new pharmacological treatment with an AM have lower use of healthcare resources as compared to previous pretreatment with AAB alone and incur lower costs in terms of concomitant medication and medical visits. This is a valuable contribution as there is a lack of information about the use of healthcare resource and costs related to the use of this combination. Few observational studies which address the use of these drugs in real life conditions have compared resource utilization before and after the addition of an AM in men on AAB therapy for the treatment of LUTS [21]. Most of the published studies are based on economic assessment models [22], the cost of treatment given [23] and/or different modalities of surgery [24].

A meta-analysis recently published by Filson and colleagues [13] compared the efficacy of combined treatment with an AAB and an AM versus monotherapy with an AAB in men with storage symptoms associated with BPH. Combination treatment was shown to be associated with significantly greater reductions in the storage symptom subscale score of the IPSS and in micturition frequency. Furthermore, this therapeutic approach was associated with only a minimal risk of either increased post-void residual volume, reduction in maximum urinary flow rate or acute urinary retention. Therefore, the authors concluded that combination therapy with AM is a reasonable treatment option for men with LUTS associated with BPH, particularly when their symptoms have a major storage component. A systematic review by Athanasopoulos et al. suggest that in men with persistent storage symptoms consistent with OAB, clinically meaningful improvements can be achieved through the addition of an antimuscarinic therapy to an a-blocker and that monotherapy with an antimuscarinic alone in this patient group is controversial, given the results of the few existing trials [25].

The results of this study supports that adding an AM agent (in combination with an AAB) leads to a reduction in the use of healthcare resources and related costs. One possible explanation could be that combination therapy provides better outcomes on storage and voiding LUTS, resulting in a reduction in the use of concomitant medication (anxiolytics, antidepressants and antibiotics) and health services (medical visits). However further investigation is required to ascertain this conclusively given the low number of patients on concomitant treatment in this study and the lack of baseline data (prior to initiating AAB treatment) of these patients. Furthermore, although there was not statistical significance in all the studied variables, a numerical reduction was observed in the majority of them. Despite these limitations, we think these findings are valuable and can help guide future investigations.

The differences in cost persisted despite the greater cost for combined treatment. In our opinion, this reduction in unit costs is important from the standpoint of efficiency in the clinical management of this group of patients, especially considering the high percentage of patients with mixed clinical symptoms with a predominance of the storage component [2]. In a meta-analysis by Xin and coworkers [26], based on 15 clinical trials (*N* = 4556), combination therapy (AAB and AM) improved LUTS with a low incidence of adverse effects. These findings are also in line with a recent meta-analysis (Gong et al.) [27] (*N* = 3063) that evaluated the efficacy and safety of tamsulosin and solifenacin combination therapy compared with tamsulosin monotherapy for male LUTS (including fixed-dose combination evidence), which concluded that this combination may be a reasonable option for male LUTS patients, especially for those who have significant storage symptoms.

It is remarkable that from the initial sample size of 26,690 subjects only 191 were on combination treatment with AAB + AM. There could be several reasons for this finding. One, only patients diagnosed with BPH according to ICPC-2 and ICD-9CM were evaluated. Additionally, during the time of the observation period for this study (2010-2012), the combination treatment with AAB + AM was already recommended in the EAU guidelines. However, the fixed dose combination, was not licensed and marketed in Spain until 2015. In contrast, the 5-ARI + AAB combination was marketed as a fixed dose combination in Spain during this time. Therefore, AAB + AM combination was recommended and could be used as a free dose combination at the time of this study. However, the extent by which the EAU recommendations were followed at this time is uncertain. None-the-less the level of use of this combination was an interesting point to assess in our study as literature about combination therapy in this profile of patients was emerging.

The limitations of the current study are those typical of retrospective studies, such as under-reporting of disease, the possible variability in clinicians’ practice and assessment of patients due to the observational design. The cost system used and/or the possible existence of a classification bias are also limitations that must be taken into account. In this regard, the possible inaccuracy of the diagnostic coding in terms of the diagnosis of BPH and other comorbidities, the reliability of the evaluation of the IPSS criteria, or the lack of a variable that might impact the final results (response to treatment, prescribed doses, etc.), must be considered.

Due to the retrospective design of the study, we cannot determine if this cost reduction is a consequence of the AM addition to the treatment with AAB or if other confounding factors could exist.

In conclusion, despite the possible limitations, this study showed that patients with LUTS associated with BPH with predominant storage symptoms who are on combination treatment with an AAB and an AM display a lower use of healthcare resources (medical visits and concomitant medication),than treatment with AAB alone,.leading to lower costs for the Spanish National Health System Further studies are warranted to determine whether the fixed-dose combination of AM + AAB (solifenacin + tamsulosin oral-controlled absorption system (TOCAS)) for the treatment of men with moderate to severe storage and voiding symptoms (available in the market after this study) reinforces or optimizes these results.

The future perspectives offered by this study could be on the potential replication of this model to better assess how treatments impact cost and resource use in other health institutions, to assist in reporting recommendations on the use of antimuscarinics to healthcare professionals.

## Conclusions

In our study, males treated with AAB monotherapy for LUTS related to BPH had a mean (SD) cost per year of 2399 (3113) € per patient. After the addition of an AM to the treatment, the mean (SD) annual cost was reduced to 2011 (3026) € per patient. The lower cost was associated with the lower use of medical visits and concomitant medication.

## Abbreviations

5ARI: 5-Alpha reductase inhibitors
AAB: Alpha-Adrenergic Blockers
ACG: Adjusted clinical groups
AM: Antimuscarinic
ANCOVA: ANalysis of COVAriance
ATC: Anatomical therapeutic chemical
BMI: Body Mass Index
BPH: Benign prostatic hyperplasia
BSA: Badalona Serveis Assistencials
CI: Confidence Interval
EAU: European association of urology
ICD-9-CM: International classification of diseases, 9th revision, clinical modification
ICPC-2: International classification of primary care
ICU: Intensive care unit
INE: Instituto Nacional de Estadística
IPSS: International prostatic symptom score
LUTS: Lower urinary tract symptoms
OR: Odds-ratio
PCC: Primary care centres
PSA: Prostatic specific antigen
RUB: Resource utilization bands
SD: Standard deviation
SPSS: Statistical product and service solutions
TOCAS: Tamsulosin oral controlled absorption system
